# Cathepsin D serum and urine concentration in superficial and invasive transitional bladder cancer as determined by surface plasmon resonance imaging

**DOI:** 10.3892/ol.2014.2250

**Published:** 2014-06-12

**Authors:** EWA GORODKIEWICZ, TOMASZ GUSZCZ, WIESLAWA ROSZKOWSKA-JAKIMIEC, ROBERT KOZŁOWSKI

**Affiliations:** 1 Department of Electrochemistry, Institute of Chemistry, University of Bialystok, Bialystok PL-15-443, Poland; 2 Department of Urology, J. Sniadecki Provincial Hospital of Bialystok, Bialystok PL-15-950, Poland; 3 Department of Analytical Chemistry, Medical University of Bialystok, Bialystok PL-15-230, Poland

**Keywords:** cathepsin D, surface plasmone resonance imaging biosensor, bladder cancer, transitional cell carcinoma

## Abstract

Determination of cathepsin D (Cat D) concentration in serum and urine may be useful in the diagnosis of bladder cancer. The present study included 54 healthy patients and 68 patients with bladder cancer, confirmed by transurethral resection or cystectomy. Cat D concentration was determined using a surface plasmon resonance imaging biosensor. Cat D concentration in the serum of bladder cancer patients was within the range of 1.3–5.59 ng/ml, while for healthy donors it was within the range of 0.28–0.52 ng/ml. In urine, the Cat D concentration of bladder cancer patients was within the range of 1.35–7.14 ng/ml, while for healthy donors it was within the range of 0.32–0.68 ng/ml. Cat D concentration may represent an efficient tumor marker, as its concentration in the serum and urine of transitional cell carcinoma patients is extremely high when compared with healthy subjects.

## Introduction

Cathepsin D (Cat D) is a ubiquitous aspartyl-family endoproteinase synthesized as a 52-kDa glycosylated preprotein, which is subsequently converted into an active two-chained (34 and 14 kDa) enzyme (1). It is distributed in lysosomes where it is involved in protein degradation and generation. Therefore, it is important for the maintenance of normal cell metabolism (2).

Previous studies have demonstrated that Cat D is involved in tumor progression. Cat D was studied in human primary breast cancer, and enzyme overexpression was found to be associated with an increased risk of metastasis and shorter survival (3,4). A similar association was identified in thyroid (5) and skin (6) cancer.

Urinary bladder cancer (UBC) is the ninth most common cancer worldwide. It is the seventh most common malignancy in males and seventeenth in females and the global standardized incidence rate is 9/100,000 in males and 2/100,000 in females (7). Annually, ~110,500 new cases in males and 70,000 new cases in females are diagnosed, and 38,200 patients in the European Union and 17,000 patients in the USA succumb to UBC (8).

Transitional cell carcinoma (TCC) biology is not completely understood. Surgical removal of the tumor mass remains the most effective treatment method. Understanding the mechanisms affecting tumor origin and progression may provide a novel theoretical basis for therapeutic methods and contribute to treatment that results in disease amelioration.

Approximately 75% of bladder cancer carcinomas are diagnosed as superficial (confined to mucosa and submucosa) and ~25% exhibit muscle-invasive disease (8).

In the present study, Cat D concentration in the serum and urine was investigated using the surface plasmon resonance imaging (SPRI) biosensor. The SPRI technique in combination with the development of sensitive biosensors is a promising tool for the determination of biologically active species. This method is label-free, easy to perform and does not require the use of radioisotopes or special substrates. The SPRI method uses an extremely specific interaction between enzymes and inhibitors (9) or antibody-antigens (10). Methods for the SPRI determination of several diagnostically significant species, including cathepsins B, D (11,12) and G (13), proteasome S20 (14), podoplanin (15) and cystatin C (16) have been developed. The SPR signal reacts to an increase in mass by changing wavelength and polarization angle. This signal is then converted to an image. Co-operation of a biosensor with the SPRI instrument ensures selectivity of the analytical signal. The biosensor contains an immobilized antibody (15) or inhibitor (9), which specifically reacts with the species to be determined. Therefore, only the species which have specifically bonded contribute to the analytical signal.

Few studies have investigated the role of Cat D in TCC. The majority of studies have focused on the evaluation of Cat D expression in TCC (17,18), and all of these studies have identified high Cat D expression in TCC tissues. Few studies have determined the concentration of various cathepsins in the serum and urine (19); however, a single study (20) reported Cat D activity in serum. The aim of this study was to determine the Cat D concentration in the blood serum and urine of patients with bladder cancer. The effects of various parameters of the urothelial cancer on the Cat D concentration were compared.

## Materials and methods

### Preparation of biological samples

Urine and serum samples of patients with bladder cancer were obtained prior to surgery or admission to the J. Sniadecki Provincial Hospital of Bialystok (Bialystok, Poland). The urine and serum samples were frozen immediately and maintained at −70°C until Cat D was analyzed. Individuals with additional malignant or inflammatory disease were excluded. Blood samples were obtained from the median cubical vein. Cancer diagnosis was detected by histological examination of tumor specimens obtained from transurethral resection or cystectomy.

Prepared serum samples were diluted two-fold with phosphate-buffered saline and transferred onto the sensor surface for 10 min. The volume of the sample applied on each measuring field was 2 μl.

Urine was centrifuged at 1,850 × g for 15 min and the supernatant was separated. Finally, the sample was filtered once through a paper filter of medium density. The prepared urine samples were then transferred onto the sensor surface for 10 min. The volume of the sample applied on each measuring field was 2 μl.

The total protein concentration was determined using Lowry’s method and creatinine (CREA) concentration was determined using Jaffe’s method.

The urine and serum concentrations of Cat D were measured in 68 patients (48 males and 20 females; mean age, 66 years) with TCC of the bladder and 54 healthy patients. Approval for this study was obtained from the Bioethics Committee of the Medical University of Bialystok (Bialystok, Poland) and written informed consent was obtained from all the patients and donors.

### Procedure of Cathepsin D determination

Cat D obtained from human liver was purchased from Sigma-Aldrich (Steinheim, Germany) and the concentration was determined using the SPRI biosensor. The SPRI technique allows sensitive determination of proteins using highly specific enzyme-inhibitor interactions. An immobilized pepstatin A (inhibitor) obtained from human liver was purchased from Sigma-Aldrich and used for the Cat D entrapment on the biosensor surface. The biosensor construction and optimization of measurement conditions used were previously described (12).

Briefly, plasma or urine samples were placed directly on the prepared biosensor for ~10 min to allow interaction with the inhibitor (pepstatin A). The biosensor was washed with water and HBS-ES buffer solution pH=7.4 (0.01 M 4-(2-hydroxyethyl)piperazine-1-ethanesulfonic acid, 0.15 M sodium chloride, 0.005% Tween 20, 3 mM EDTA) (all Biomed-Lublin, Lublin, Poland) to remove unbound molecules from the surface. The SPRI signal was measured twice on the basis of registered images, following the immobilization of pepstatin A and then following interaction with Cat D from the samples. The signal, which is proportional to coupled biomolecules, was obtained by calculating the difference between the signal prior to and following the interaction with biomolecules. The concentration was determined using the calibration curves of the SPRI signal depending on the concentration of Cat D.

### Statistical analysis

The results are presented as the median ± standard deviation. Statistical analyses were performed using Student’s t-test, and P<0.05 and P<0.01 were considered to indicate a statistically significant difference.

## Results

### Changes in Cat D concentration

**Figure 1:**
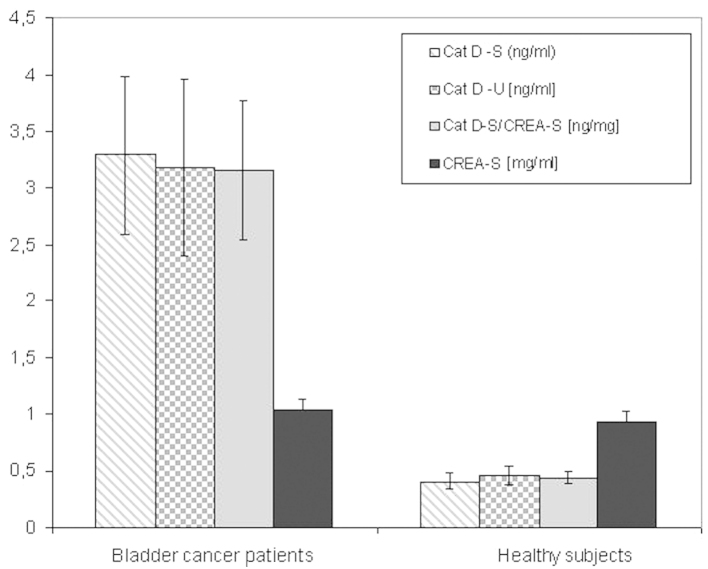
Concentration of Cat D in blood serum and urine, as well as concentration of CREA in blood serum, of bladder cancer patients and healthy subjects. The Cat D/CREA serum ratio is presented for comparison. Confidence bars were calculated at P=0.01. Cat D, cathepsin D; CREA, creatinine; S, serum; U, urine.

The Cat D concentration in serum (Table I) and urine (Table II) samples was investigated with regard to the bladder cancer parameters. Cat D/total protein and Cat D/CREA ratios are also shown in Tables I and II. A summary of the results are presented in Fig. 1.

A significant difference in serum and urine Cat D concentration levels was observed between bladder cancer patients and healthy subjects (Fig. 1). This indicates the potential of Cat D as a cancer marker. No significant differences in CREA concentration were identified between bladder cancer patients and healthy subjects. To further investigate the results of the present study, Cat D concentration was corrected by serum CREA concentration to eliminate the impact of renal impairment on the observed results. Furthermore, the Cat D/protein ratio was introduced as a novel parameter. In this way, one of the causes of inflammatory proteinuria was eliminated.

### Blood serum analysis

In terms of different cancer parameters, few parameters in the serum were statistically significant. When comparing invasive and superficial tumors, values were almost identical; however, the Cat D/CREA ratio was found to be significantly higher in superficial tumors when compared with invasive tumors (P<0.05; Table I). This was due to the significantly higher CREA concentrations (data not shown) identified in invasive tumors when compared with superficial tumors (P<0.05).

In recurrent, multifocal, high-grade and smaller (<30 mm) tumors, serum Cat D levels were elevated; however, no significant differences were identified. This pattern was confirmed by the Cat D/CREA ratio in all the aforementioned groups. Males and older individuals were characterized by higher levels of Cat D; however, this difference was not statistically significant and did not confirm this correlation in relation to the Cat D/CREA ratio (Table I).

### Urine analysis

In urine, a significantly higher Cat D/protein ratio was demonstrated in primary, single, smaller and low-grade groups of cancer. Notably, in the case of low-grade tumors, Cat D/protein ratio was significantly higher than that of high-grade tumors, while Cat D concentration alone was marginally elevated in high-grade tumors. This may be explained by the significant difference in protein concentration (data not shown) identified between low- and high-grade tumors (P<0.05).

## Discussion

The majority of UBCs are TCCs. The effect of various parameters of TCCs on Cat D concentrations were analysed in this study. The most significant result of the present study is that all bladder tumor cases exhibited significantly higher serum (eight-fold) and urine (seven-fold) Cat D concentrations when compared with healthy control subjects. This shows the efficacy of Cat D concentration as a tumor marker. In the serum, the lowest Cat D concentration for TCC was 1.3 ng/ml, whereas the highest Cat D concentration for healthy donors was 0.52 ng/ml. In the case of urine, the lowest Cat D concentration for TCC was 1.35 ng/ml, whereas the highest Cat D concentration for healthy donors was 0.68 ng/ml. This comparison shows that the concentration of Cat D may have prognostic value for excluding TCC, and Cat D may be used as a tumor marker to reduce the number of cystoscopies.

TCC patients were found to exhibit extremely high, but relatively stable, levels of serum and urine Cat D, which were independent of tumor parameters. Serum Cat D concentrations were found to range between 1.30 and 5.59 ng/ml with the majority of the results at ~3.3 ng/ml and, in the case of urine, the concentration was found to range between 1.35 and 7.14 ng/ml with the majority of the results at ~3.2 ng/ml.

A high recurrence rate is characteristic of TCC of the bladder. In superficial stages, Ta and T1, as well as in particular cases of T2a, it may be effectively cured by bladder-sparing treatment (21). Effectively controlling bladder TCC prolongs survival; however, this requires strict follow-up procedures to guarantee early detection. The European Association of urology (22) and American Association of Urology (23) consistently recommend performing cystoscopy with established procedures. Previous studies have attempted to identify a tumor marker in the blood or urine to facilitate diagnosis and eliminate invasive procedures (24,25). Urine cytology, which is recognized as a traditional test, has low sensitivity. Therefore, a negative result does not exclude the patient from obligatory cystoscopy (26). Novel substances are verified as potential highly sensitive markers to reduce the number of cystoscopies (27).

Further studies using larger numbers of patients are required, which investigate the association between Cat D and the individual parameters that characterize bladder cancer, in particular the recurrence and prediction of progression.

## Figures and Tables

**Table I:** Diagnostic characteristics of serum Cat D/protein and Cat D/CREA concentration ratios compared with various parameters of urothelial cancer.

		Cat D/protein (ng/nl)			Cat D/S-CREA (ng/ml)		
					
Parameter	n	Range	Mean ± SD	P-value	Range	Mean±SD	P-value
Primary/recurrent				NS			NS
Primary	33	0.037–0.072	0.059±0.030		1.65–3.85	2.79±1.33	
Recurrent	35	0.028–0.089	0.062±0.024		2.45–4.29	3.63±1.06	
Multiplicity				NS			NS
Single	22	0.041–0.086	0.056±0.023		1.98–4.13	3.04±1.61	
Multiply	46	0.038–0.089	0.065±0.022		2.04–4.62	3.39±1.23	
Stage				NS			<0.05
Superficial (Ta + T1)	47	0.027–0.089	0.060±0.025		2.76–5.11	3.86±1.56	
Invasive (T2 + T3=T4)	21	0.048–0.079	0.066±0.020		1.64–3.69	2.27±1.12	
Grade				NS			NS
Low-grade	22	0.027–0.072	0.057±0.018		1.06–4.30	3.17±1.43	
High-grade	46	0.041–0.089	0.065±0.028		1.14–4.89	3.23±1.79	
Size (mm)				NS			NS
<30	39	0.038–0.098	0.062±0.020		2.59–5.04	3.47±1.28	
>30	29	0.038–0.087	0.059±0.028		1.78–4.98	2.87±1.74	
Gender				NS			NS
Female	20	0.038–0.098	0.056±0.017		1.67–4.85	3.35±1.20	
Male	48	0.038–0.073	0.059±0.028		1.85–5.13	3.12±1.50	
Age (years)				NS			NS
<65	35	0.027–0.089	0.056±0.022		2.60–5.05	3.24±1.06	
≥65	33	0.038–0.085	0.065±0.019		2.87–5.98	3.26±1.94	

Total protein concentration was determined by Lowry’s method and creatinine concentration was determined by Jaffe’s method. Cat D, cathepsin D; S-CREA, serum creatinine; SD, standard deviation; NS, not significant.

**Table II:** Diagnostic characteristics of urine protein, Cat D and Cat D/protein ratio compared with various parameters of urothelial cancer.

		Cat D (ng/ml)			Cat D/protein (ng/mg)		
					
Parameter	n	Range	Mean±SD	P-value	Range	Mean±SD	P-value
Primary/recurrent				NS			<0.01
Primary	33	2.51–4.35	3.41±0.95		5.25–12.5	9.48 ±2.28	
Recurrent	35	2.25–5.31	3.09±0.93		2.68–7.28	4.23±1.19	
Multiplicity				NS			<0.01
Single	22	2.42–5.19	3.46±1.01		5.60–9.29	8.65±1.35	
Multiply	46	2.15–5.31	2.90±0.94		4.13–8.36	6.59±1.68	
Stage				NS			NS
Superficial (Ta+T1)	47	2.90–5.85	3.21±0.76		3.90–11.60	7.64±2.72	
Invasive (T2+T3=T4)	21	1.35–7.14	3.57±1.80		4.85–9.10	6.15±1.69	
Grade				NS			<0.01
Low-grade	22	2.42–4.35	3.41±0.70		8.96–16.10	13.37±2.72	
High-grade	46	1.79–6.32	3.45±1.26		7.16–10.70	8.02±1.42	
Size (mm)				NS			<0.01
<30	39	1.85–7.14	3.69±1.49		9.20–14.20	11.8±1.75	
>30	29	2.55–6.32	3.52±1.14		6.43–12.15	9.78±2.14	
Gender				NS			NS
Female	20	1.89–6.32	3.77±1.59		7.37–11.15	8.77±1.29	
Male	48	2.42–7.14	3.51±1.20		6.09–10.80	8.36±1.69	
Age (years)				NS			<0.05
<65	35	1.89–7.14	3.39±1.43		6.91–14.50	9.68±2.89	
≥65	33	2.55–6.32	3.84±1.14		5.90–9.10	7.38±1.69	

Total protein concentration was determined by Lowry’s method. Cat D, cathepsin D; SD, standard deviation; NS, not significant.
